# Widespread GLI expression but limited canonical hedgehog signaling restricted to the ductular reaction in human chronic liver disease

**DOI:** 10.1371/journal.pone.0171480

**Published:** 2017-02-10

**Authors:** Candice Alexandra Grzelak, Nicholas David Sigglekow, Janina Elke Eleonore Tirnitz-Parker, Elizabeth Jane Hamson, Alessandra Warren, Bharvi Maneck, Jinbiao Chen, Bramilla Patkunanathan, Jade Boland, Robert Cheng, Nicholas Adam Shackel, Devanshi Seth, David Geoffrey Bowen, Luciano Gastón Martelotto, D. Neil Watkins, Geoffrey William McCaughan

**Affiliations:** 1 Liver Injury and Cancer, Centenary Institute, Camperdown, New South Wales, Australia; 2 School of Biomedical Sciences, Curtin Health Innovation Research Institute, Curtin University, Bentley, Western Australia, Australia; 3 Ageing and Alzheimers Institute, Centre for Education and Research on Ageing, University of Sydney, Concord Hospital, Sydney, New South Wales, Australia; 4 ANZAC Research Institute, University of Sydney, Concord Hospital, Sydney, New South Wales, Australia; 5 A.W. Morrow Gastroenterology and Liver Centre, R.P.A.H., Camperdown, New South Wales, Australia; 6 Faculty of Medicine, University of Sydney, Sydney, New South Wales, Australia; 7 Department of Pathology, Memorial Sloan Kettering Cancer Center, New York, New York, United States of America; 8 The Kinghorn Cancer Centre, Garvan Institute, Darlinghurst, New South Wales, Australia; Vrije Universiteit Brussel, BELGIUM

## Abstract

Canonical Hedgehog (Hh) signaling in vertebrate cells occurs following Smoothened activation/translocation into the primary cilia (Pc), followed by a GLI transcriptional response. Nonetheless, GLI activation can occur independently of the canonical Hh pathway. Using a murine model of liver injury, we previously identified the importance of canonical Hh signaling within the Pc^+^ liver progenitor cell (LPC) population and noted that SMO-independent, GLI-mediated signals were important in multiple Pc^-ve^ GLI2^+^ intrahepatic populations. This study extends these observations to human liver tissue, and analyses the effect of GLI inhibition on LPC viability/gene expression. Human donor and cirrhotic liver tissue specimens were evaluated for SHH, GLI2 and Pc expression using immunofluorescence and qRT-PCR. Changes to viability and gene expression in LPCs *in vitro* were assessed following GLI inhibition. Identification of Pc (as a marker of canonical Hh signaling) in human cirrhosis was predominantly confined to the ductular reaction and LPCs. In contrast, GLI2 was expressed in multiple cell populations including Pc^-ve^ endothelium, hepatocytes, and leukocytes. HSCs/myofibroblasts (>99%) expressed GLI2, with only 1.92% displaying Pc. *In vitro* GLI signals maintained proliferation/viability within LPCs and GLI inhibition affected the expression of genes related to stemness, hepatocyte/biliary differentiation and Hh/Wnt signaling. At least two mechanisms of GLI signaling (Pc/SMO-dependent and Pc/SMO-independent) mediate chronic liver disease pathogenesis. This may have significant ramifications for the choice of Hh inhibitor (anti-SMO or anti-GLI) suitable for clinical trials. We also postulate GLI delivers a pro-survival signal to LPCs whilst maintaining stemness.

## Introduction

Cirrhosis as a result of chronic liver disease (CLD) is a major cause of worldwide morbidity and mortality [1]. Liver transplantation remains the only curative treatment option. Further, a lack of suitable donors renders this option implausible for the majority of patients. Therefore, novel therapeutic approaches for the successful treatment of CLD are in high demand.

To develop such approaches, it is essential to elucidate the cellular biology that underpins the regulation of pathogenic liver microenvironmental niches. A key feature of chronic human and mouse liver disease pathology [2,3] is the ductular reaction, thought to include expansion of putative bipotential liver progenitor cells (LPCs) [4–6]. We were the first to describe that primary cilia (Pc) are present on LPCs in mouse liver tissue [7]. Pc are single, non-motile, membrane-bound cellular organelles, essentially an ‘antennae’ (reviewed by [8]) that receive extracellular environmental cues (e.g. ligand) and translate these cues into internal cellular outputs (e.g. transcriptional response).

In vertebrate cells, canonical Hh signals are transduced through the Pc [9–11]. This occurs following Hh ligand binding (Sonic Hh, SHH; Indian Hh, IHH; or Desert Hh, DHH) to its receptor PATCHED1. This action de-represses SMOOTHENED (SMO), facilitating the activation/translocation of SMO into the Pc. Consequently, the GLI family (GLI1, GLI2, GLI3) of transcription factors are stabilized into their transcriptionally active forms, driving GLI target gene expression [12]. This is known as canonical Hh/Pc/SMO/GLI-mediated signaling. However, GLI signals can also be driven *independently* of Pc/SMO. This is referred to as Pc/SMO-independent GLI-mediated signaling, recently reviewed [12].

A thorough, accurate assessment of the intrahepatic cell populations contributing to Pc/SMO-dependent GLI-mediated signaling and Pc/SMO-independent GLI-mediated signaling in CLD is imperative, due to the therapeutic potential of SMO small molecule inhibitors in the treatment of CLD—currently in stage I clinical trials [13]. A major limitation of former studies is the assumption that GLI2^+^ cells are ‘actively’ signaling via a canonical Hh pathway [14–18]. As we have previously asserted, this is a misleading assumption [7,19,20].

In the experimental thioacetamide (TAA) model of cirrhosis, we recently demonstrated that canonical Hh/Pc/SMO/GLI-mediated signaling amplifies the LPC response [7]. Further, our results suggest that GLI signaling is mediated by at least two mechanisms within intrahepatic populations in mouse liver tissue—Pc/SMO-dependent and Pc/SMO-independent GLI-mediated signaling. However, it is plausible our observations apply specifically to a single model of experimental liver disease. Importantly, cell populations signaling via a Pc/SMO-independent GLI-mediated mechanism would not be directly targeted using anti-SMO therapy; these populations may instead benefit from anti-GLI therapy.

To address the limitations of former human studies, and potentially reconcile these with our own observations, in this paper we specifically addressed: 1) Whether canonical Hh/Pc/SMO/GLI-mediated signaling participates in regulation of the human LPC niche *in vivo*; 2) Clarification of whether GLI-mediated signaling is driven through Pc/SMO in other intrahepatic cell populations in human CLD; 3) The contribution of GLI inhibition to LPC viability, proliferation and gene expression in culture. Our results have direct therapeutic implications for the design of human clinical CLD trials involving Hh inhibitors.

## Materials and methods

### Patient samples

Ethics for human liver tissue collection were firstly submitted to, and approved by, the Human Research Ethics Committee, Sydney Local Health District, RPAH Zone. Human liver tissue was collected from consenting patients according to methods outlined in approved X10-0072 protocol. Participants provided written informed consent. Samples included representative donor (*n* = 5) and cirrhotic samples from various disease etiologies (alcoholic liver disease (ALD) *n* = 6; non-alcoholic steatohepatitis (NASH) *n* = 3; primary biliary cirrhosis (PBC) *n* = 1). Patient age, sex, and fibrosis staging (Scheuer score [21]) are provided in Table 1.

**Table 1:** Patient sample, age, sex, fibrosis staging (Scheuer score) and SHH expression.

Tissue	Age (time tx)	Sex	Fibrosis stage	SHH: hepatocyte	SHH: non-parenchymal	SHH+ EpCAM+
donor	54	F	F = 0	Negligible	Yes	Yes
donor	23	F	F = 0		Yes	Yes
donor	64	M	F = 0		Yes	Yes
donor	83	M	F = 0		Yes	Yes
donor	60	M	F = 0		Yes	Yes
ALD	62	M	F = 4	E-M interface	Yes	n/a
ALD	60	M	F = 4	E-M interface	Yes	n/a
ALD	62	M	F = 4	Widespread: nodule + interface	Negligible	Yes
ALD	45	M	F = 4	Widespread: nodule + interface	Yes	Yes
ALD	47	M	F = 4	E-M interface	Yes	n/a
ALD	56	M	F = 4	High expression E-M interface	Yes	Weak
NASH	64	M	F = 4	Widespread: nodule + interface	Yes	Weak
NASH	52	M	F = 4	High expression E-M interface	Yes	Yes
NASH	67	M	F = 4	High expression E-M interface	Yes	Yes
PBC	60	M	F = 3	Low expression	Yes	Yes

Abbreviations: Tx, transplant; ALD, alcoholic liver disease; NASH, non-alcoholic steatohepatitis; PBC, primary biliary cirrhosis; E-M, epithelial-mesenchymal interface

### Immunofluorescence (IF)

Frozen human liver tissue was cryosectioned (4 μm). For Pc IF, sections were fixed in 10% neutral buffered formalin (NBF) for 20 min at room temperature (RT). Following washing (3 x 10 min, 1xPBS), permeabilisation (0.5% (v/v) TX-100/PBS, 20 min, RT), and subsequent washing, blocking was performed using 10% normal serum diluted in Odyssey buffer (LI-COR) for 1 h at RT or 4°C overnight. For SHH and GLI2 IF, sections were fixed in 10% NBF for 30 min prior to antigen retrieval using pre-heated 1x Universal Decloaker (Biocare Medical) in a water bath (4.5 min, 92°C). Slides were cooled to RT followed by washing and blocking steps as outlined above. Primary antibodies were diluted in Odyssey buffer + 0.1% (v/v) TX-100 and incubated for 2 h at RT (Pc, SHH) or 4°C overnight (GLI2). Primary antibodies include α-acetylated tubulin (1:10, mouse, T6074, Sigma-Aldrich), γ-tubulin (1:200, rabbit, C7604, Sigma-Aldrich), Arl13b (1:100, rabbit, 17711-1-AP, Proteintech), CD31 (1:20, mouse, M0823, Dako), CD45 (1:100, mouse, C7556, Sigma-Aldrich), CK18 (1:50, mouse, M7010, Dako), EpCAM (1:50, mouse, MS-144-P, Thermo Scientific), GLI2 (1:100, rabbit, ab7181, Abcam), ki67 (1:100, mouse, BDB561126 or 1:200, rabbit, 1:200, ab15580), pan-cytokeratin (pan-CK, 1:400, rabbit, Z0622, Dako), SHH (1:600, ab53281, Abcam), and Vimentin (1:200, rat, MAB2105, R&D). Alternatively, sections were incubated with appropriate isotype controls (mouse IgG_1κ_ 557273, BD Pharminogen; rabbit IgG 026102, rat IgG_2a_ 029688, Life Technologies) at the same final concentration as the primary antibody (S1 Fig). Primary cilium antibodies were directly conjugated to fluorophores (α-acetylated tubulin-CF-488A, Mix-n-Stain, Biotium; γ-tubulin-Cy3, Sigma-Aldrich). Secondary antibodies (1:400, Alexa Fluor, Life Technologies) were incubated for 45 min at RT. Slides were mounted using ProLong Gold + DAPI.

### Immunocytochemistry

Cells (BMOL1.2, BMOL-TAT) were plated at ~70% sub-confluency in chamber slides (Sarstedt). Once reaching confluence, following a rinse in PBS, cells were fixed in 10% neutral buffered formalin for 20 min at RT. Following washing (3 x 5 min, 1x PBS), permeabilisation (0.5% (v/v) TX-100/PBS, 20 min RT), and another wash, blocking was performed using Odyssey buffer (LI-COR) for 1 h, RT. Primary antibodies were incubated for 2 h at RT and are listed above, excepting Osteopontin (AF808, R&D). Fluorescent signal was detected using secondary antibodies or direct conjugation to CF dyes, as above.

### Imaging

Screening of human liver samples for SHH and GLI2 was initially performed using a Leica DM6000B. Identical exposure conditions were used for imaging the protein of interest and matching IgG control (S1 Fig). Confocal imaging was performed using a Leica SP5. For Pc screening, *n* = 8–10 fields of view (FOV) were imaged through the fibrotic septae where Pc^+^ cells were located (*n* = 3 ALD samples, Pc/LPC marker (EpCAM or pan-CK), Pc/Vimentin, Pc/CD31; *n* = 1 ALD sample Pc/CD45). All Pc images were obtained using the following imaging parameters: 63x objective, 1.5X digital zoom, 6x 0.7μm steps, 1024x1024 pixels. All Pc images represent a maximum intensity projection (MIP); quantification was performed on the MIP. For Pc quantitation, only fully formed Pc (axoneme—comprising α-acetylated tubulin—assembled from the basal body comprising γ-tubulin) were counted. To determine the % of LPCs expressing Pc, a total of 566 Pc and 1259 LPC cells (either EpCAM^+^ or pan-CK^+^) were counted. To determine the % of vimentin^+^ hepatic stellate cells (HSCs) expressing Pc, a total of 510 Pc and 3375 vimentin^+^ cells were counted.

A triple stain for EpCAM, vimentin and GLI2 was performed to quantitate the % of GLI2^+^ LPCs and GLI2^+^ HSCs within the same FOV. Images (ALD *n* = 3, *n* = 8 FOV/sample) were obtained through the fibrotic septae, using identical imaging parameters. A total of 1129 EpCAM^+^ cells and 1902 Vimentin^+^ cells were counted. Cells were considered GLI2^+^ if they expressed nuclear GLI2.

### Electron microscopy

Human liver tissue (~1 mm^3^) was washed in PBS prior to placing in fixative (2% (v/v) glutaraldehyde 1% (w/v) sucrose PBS, pH 7.4) O/N 4°C. BMOL1.2 and BMOL-TAT were grown on thermanox coverslips and fixed for 1 h RT in 4% Glutaraldehyde-EM-grade in PBS pH 7.4 to equal amounts of medium and mixed gently. For scanning electron microscopy (SEM) cells were osmicated (1% OsO_4_/0.1 mol/l sodium cacodylate buffer), dehydrated in an ethanol gradient to 100%, and incubated for 2 min in hexamethyl-disilazane. Coverslips were then mounted on stubs, sputter coated with platinum, and examined using a JEOL 6380 SEM. For transmission electron microscopy (TEM), coverslips and tissue samples were embedded in Spurr's resin following the post fixation and dehydration steps. Ultrathin sections were obtained and viewed on a Phillips CM10 TEM.

### Quantitative PCR

Total RNA was extracted from liver tissues with TRIzol (Life Technologies). cDNA was generated from total RNA with high capacity reverse transcription kits (Life Technologies). The abundance of transcripts was assessed by quantitative real-time PCR (qPCR) on a Corbett Rotor-Gene 3000 with SsoFast EvaGreen (Bio-Rad). The level of each transcript was determined by a standard curve. Expression of *SHH*, *GLI1* and *GLI3* was normalised for the efficiency of amplification with both *PPIA* and *ACTB*. The sequences of primer pairs used in qPCR were PPIA sense GGCAAATGCTGGACCCAACACA and PPIA antisense CTAGGCATGGGAGGGAACAAGG; ACTB sense ATTGGCAATGAGCGGTTC and ACTB antisense GGATGCCACAGGACTCCAT; SHH sense AGTTTCACTCCTGGCCACTG and SHH antisense GATGAAGAAAACACCGGAGC; GLI1 sense TAGCTACTGATTGGTGGTGGG and GLI1 antisense ACTCCAGCCCTGGACCG; GLI3 sense GGCTGCATAGTGATTGCGT and GLI3 antisense CGAACAGATGTGAGCGAGAA.

### Western blot

Protein from donor (n = *3* samples) and ALD (n = *3* samples) frozen liver was extracted using RIPA buffer containing protease and phosphatase inhibitors (Roche). Proteins were resolved on a 4–12% NuPAGE Bis-Tris gel, transferred to polyvinylidene difluoride (PVDF) membranes (Millipore, Billerica, MA), and then blocked with 10% (w/v) skim milk powder in PBS for 1 h at RT. The GLI1 rabbit polyclonal (1:2000, sc-20687, Santa Cruz Biotechnology) and GAPDH monoclonal (1:10,000, #ab8245, Abcam) antibodies were incubated with the membranes for 16 h at 4°C. After being washed three times, the membranes were incubated with horseradish peroxidase conjugated secondary antibodies for 1 h at RT. After washing three times with PBS, the membrane was incubated with Pierce ECL Western blotting substrate (Thermo Fisher Scientific). Blots were quantified with NIH Image J and normalised by GAPDH.

### Cell culture

BMOL1.2 and BMOL-TAT cells were obtained from Dr. Tirnitz-Parker [22] and cultured as previously described [7]. Experiments were performed between passages 2–6 after revival from N_2_(*l*). Cell lines were confirmed negative for mycoplasma using the MycoAlert Detection Kit (Lonza).

### Viability assays

Cells (2000/well) were seeded into 96-well plates. GANT61 (10 μM), DAPT (10 μM), SGX523 (500 nM) or XAV939 (1 μM) and equivalent vehicle (DMSO) were added to sextuplicate wells. Viability was assessed by fluorescence (FLUOstar Omega) using ALAMAR blue (Life Technologies). Experiments were repeated sequentially a minimum three times for each cell line. Two-way ANOVA with Tukey’s correction for multiple comparison (α = 0.001) was performed (GraphPad Prism 6).

### RT^2^ PCR profiling arrays

Cells (5x10^5^) were plated into 10cm dishes, and medium replaced with 0.2% FBS culture medium for 16 h prior to treatments. Cells were treated with GANT61 (10 μM) or DMSO equivalent in 0.2% FBS for 8 h. Cells were then washed with 1xPBS, and RNA isolated on ice by scraping and RNeasy kit (Qiagen). The experiment was repeated in triplicate per line. cDNA was generated from RNA (1 μg) using the RT First Strand Synthesis kit (Qiagen). Custom 384-well murine gene arrays (CAPM12471E; S1 Table) were loaded using an EpMotion robot and qPCR carried out using an ABI-7900HT (Applied Biosystems) at the ACRF Facility, Garvan Institute. Controls included PPC, RTC, and MGDC wells, four constitutive gene expression controls (*gapdh*, *hsp90ab1*, *b2m*, *rn18s*), and the cycle threshold set to ensure a consistent PPC value. Analysis using online SABioscience PCR Array software was performed for each individual experiment, cell line (*n* = 3) and combination of LPC lines (*n* = 6). Data were standardised to *b2m* and *gapdh* and a 1.5 fold-change set for gene transcript comparison. Consistent gene changes were generated from *n* = 3 analyses, and included only genes that modulated in the same direction in ≥2/3 experiments. Data were converted to log_2_ scale and displayed in heat-map format using GENE-E.

## Results

### Hh-producing cells in human donor and cirrhotic tissue

**Fig 1:**
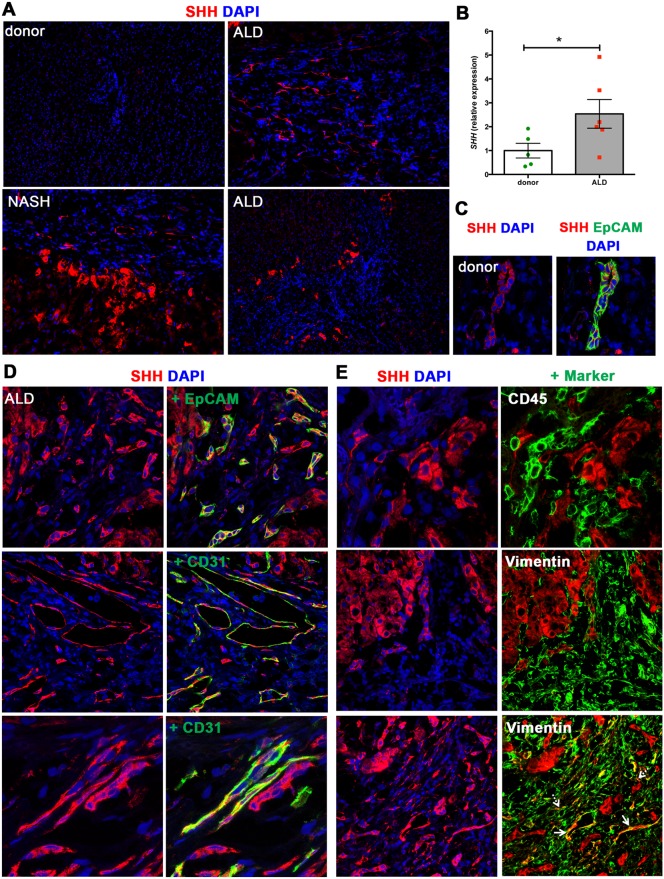
SHH expression in human donor and cirrhotic liver. **(A)** Frozen (4 μm) human donor (*n* = 5), and cirrhotic liver sections [ALD (*n* = 6), NASH (*n* = 3), PBC (*n* = 1)] were screened for SHH (C-terminus) by immunofluorescence. 5x objective. Representative images shown. **(B)** qRT-PCR for *SHH* in human donor and ALD samples. Mean±S.E.M. Significant (*) difference between means (One-sided student t-test, *p<0.05). **(C)** SHH expression (red) by EpCAM^+^ LPCs (green) in donor liver. 63x objective. **(D, E)** Comprehensive characterisation of SHH expression by liver cell populations in ALD. Images obtained using 63x objective. The majority of SHH is produced by hepatocytes. **(D)** SHH (red) is expressed by EpCAM^+^ LPCs (green). Co-localisation in merged images indicated by yellow. A subset of CD31^+^ ECs (green) at the cirrhotic interface express SHH. **(E)** SHH (red) is not expressed by CD45^+^ leukocytes (green). Minimal SHH is expressed by vimentin^+^ ECs (solid arrows), with negligible SHH expressed by vimentin^+^ myofibroblasts (dashed arrows). DAPI, blue.

We firstly established the cellular source of Hh ligand in donor and cirrhotic human liver tissue specimens. In line with previous studies [23], we observed minimal SHH ligand expression (C-terminal) in donor liver samples (Table 1, Fig 1A; *n* = 5 samples, representative image displayed). However, in chronically injured liver [ALD *n* = 6, F = 4 (cirrhosis, Scheuer score) NASH *n* = 3, F = 4] hepatocytes displayed strong SHH positivity (Fig 1A; S2A Fig, representative images) [23]. Varying patterns of SHH expression were seen, outlined in Table 1. Importantly, the majority of Hh ligand production in human liver disease tissue appeared to be derived from hepatocytes, located at the epithelial-mesenchymal interface or within the regenerative nodule. High levels of *SHH* in ALD (vs. donor) were also confirmed at the mRNA level (Fig 1B).

Interestingly, we noticed rare SHH^+^ cells around the portal tracts in donor human liver. These were confirmed to be EpCAM^+^ and were present in all donor samples (*n* = 5) screened (Table 1, Fig 1C) but not observed in the mouse model [7]. Notably, the EpCAM^+^ structures were observed as pseudo-ductular strings [24], indicative of the cellular component of the ductular reaction—a reactive process that is induced at the portal/parenchymal interface in chronically injured livers and that encompasses proliferation of bipotential LPCs amongst inflammatory, neural, vascular and extracellular matrix changes [25]. Further, EpCAM^+^ SHH^+^ ductular reactions were also noted at the epithelial-mesenchymal interface in ALD (Fig 1D).

Endothelial cells (ECs) [26], leukocytes [27] and HSCs [28] are reported to contribute to Hh ligand production in cirrhotic liver disease. Hence, we examined whether CD31^+^ ECs, CD45^+^ leukocytes or vimentin^+^ HSCs/myofibroblasts expressed SHH (C-terminal). Unlike our animal model [7], a portion of CD31^+^ vascular ECs (within larger venous structures) expressed SHH (Fig 1D), whereas CD45^+^ leukocytes did not. Of note, minimal expression of SHH was detected within the vimentin^+^ cell population. The cellular source of SHH ligand appeared to be vascular ECs (which co-express vimentin and CD31; Fig 1E, solid arrows) rather than vimentin^+^ myofibroblasts (Fig 1E, dashed arrows).

### GLI2 expressing cell populations in human donor and cirrhotic tissue

**Fig 2:**
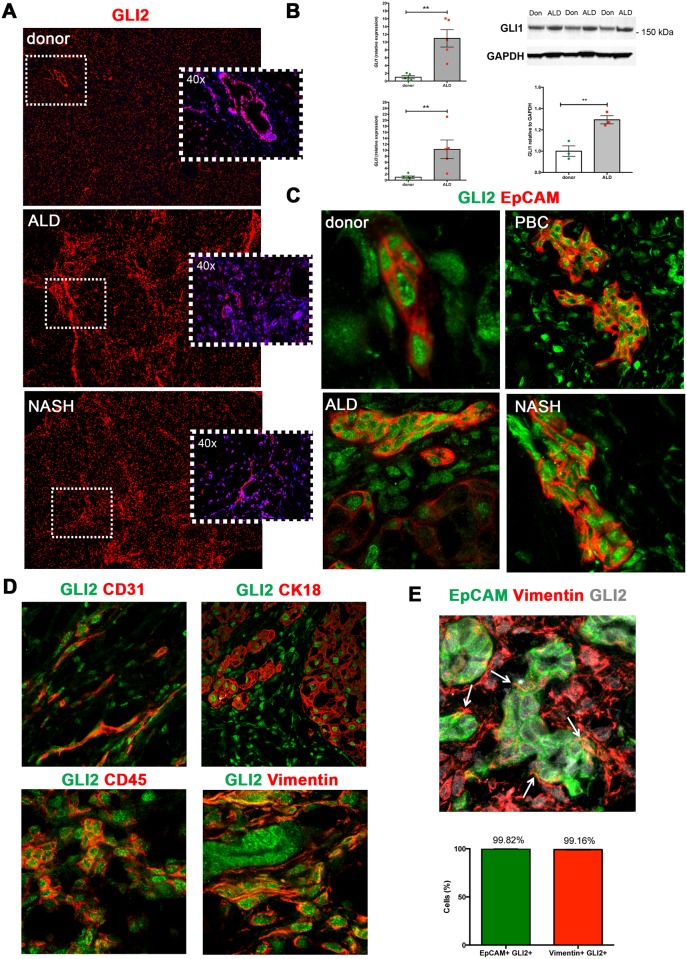
Widespread GLI expression in human donor and cirrhotic liver. **(A)** Frozen (4 μm) human donor (*n* = 5), and cirrhotic liver sections [ALD (*n* = 6), NASH (*n* = 3), PBC (n = 1)] were screened for GLI2 (red) expression by immunofluorescence. Representative images taken at 5x or 40x (insets) objective shown. DAPI, blue. **(B)** qRT-PCR for *GLI1* and *GLI3* transcript in human donor or ALD samples. Mean±S.E.M. Significant (*) difference between means (One-sided student t-test, **p<0.005). Western blot for full-length GLI1 protein (>150 kDa) in donor (Don) or ALD patient samples. Densitometry analysis with GLI1 normalised to GAPDH (Image J). Mean±S.E.M; **p = 0.0093 (Two-sided student t-test). **(C)** Nuclear GLI2 (green) expression in EpCAM^+^ (red) LPCs in donor, ALD, PBC and NASH liver. **(D)** Nuclear GLI2 (green) expression demonstrated within CD31^+^ (red) ECs, CK18^+^ (red) hepatocytes, CD45^+^ (red) leukocytes and vimentin^+^ (red) HSCs/myofibroblasts, in ALD. 63x objective. **(E)** Maximum intensity projection illustrating close physical association between EpCAM^+^ LPCs (green) and vimentin^+^ HSCs/myofibroblasts (red), both of which express GLI2 (grey), in ALD tissue. Arrows indicate myofibroblasts directly contacting LPCs. Confocal microscopy, 63x objective. Quantitation (%) of EpCAM^+^ GLI2^+^ cells and vimentin^+^ GLI2^+^ cells within the same FOV (*n* = 3 ALD samples, 8 FOV/sample).

Next, we sought to identify potential Hh-responsive cells using GLI2, the marker used in all prior studies of Hh signaling within a liver disease context [14,16]. Upon screening for GLI2 in our cohort of human donor and cirrhotic liver samples, we found GLI2 to be widely expressed within the lobule during liver homeostasis (Fig 2A, *n* = 5 donor; representative image). The number of GLI2^+^ cells was found to increase during human cirrhotic injury (ALD *n* = 6, NASH *n* = 3, PBC *n* = 1) (Fig 2A; representative images shown). In a way akin to the TAA model, *GLI1* and *GLI3* transcripts were found ~10-fold increased in ALD compared to donor liver tissue (Fig 2B). GLI1 protein was also significantly increased in ALD vs. donor liver tissue (*p* = 0.0093), albeit to a lesser degree (~0.3-fold increase) than transcript (Fig 2B). Importantly, GLI2 was readily expressed within the EpCAM^+^ ductular/LPC population in donor, ALD, PBC and NASH liver specimens (Fig 2C, representative images).

Furthermore, and in line with the TAA model, GLI2^+^ cell populations included CD31^+^ ECs, CK18^+^ hepatocytes, CD45^+^ leukocytes and vimentin^+^ HSCs/myofibroblasts (Fig 2D) [7]. Within the same FOV, GLI2^+^ vimentin^+^ HSCs/myofibroblasts were found intimately associated and physically intertwined with GLI2^+^ EpCAM^+^ ductular/LPC cells in ALD tissue (Fig 2E). Importantly, 99.82% of EpCAM^+^ ductular/LPC cells and 99.16% vimentin^+^ HSCs were GLI2^+^ in ALD tissue (Fig 2E).

### Identification of Pc on the human ductular reaction/LPCs

**Fig 3:**
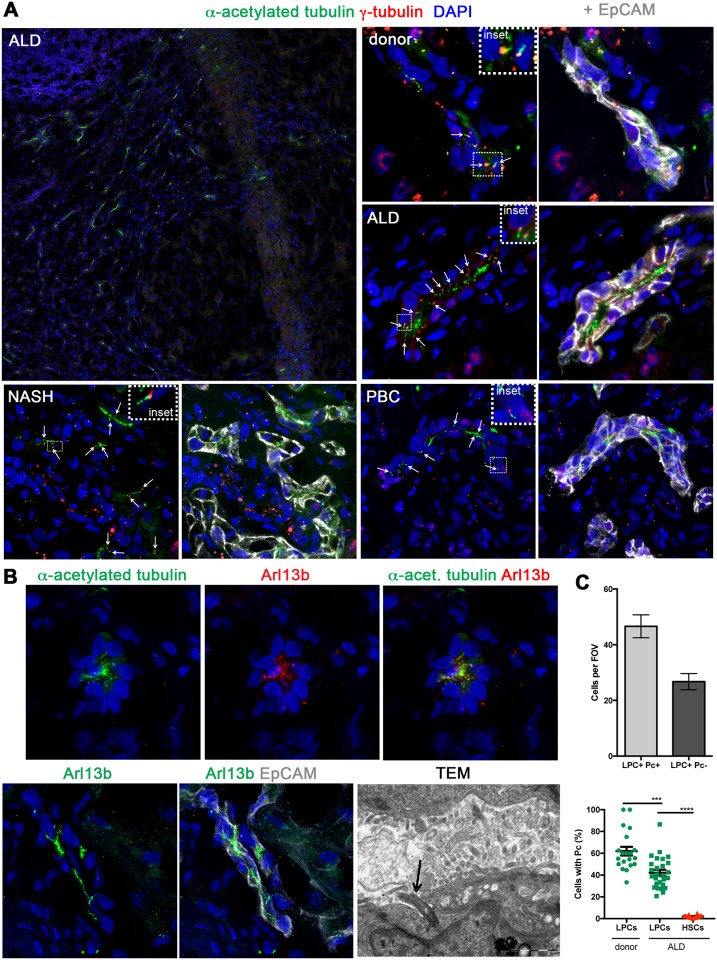
Primary cilium expression in human EpCAM^+^ ductular reaction and liver progenitor cells *in vivo*. **(A)** Immunofluorescence identified primary cilia (Pc) in human ALD tissue at the injury interface. Fully assembled Pc are identified by extension of an axoneme (α-acetylated tubulin, green) from the basal body (γ-tubulin, red). Pc were identified (white arrows) on EpCAM^+^ (grey) ductular reaction/LPCs in human donor liver and in a range of cirrhotic liver tissues (ALD, NASH, PBC). **(B)** In human ALD tissue, α-acetylated tubulin (green) co-localised with Arl13b (red), confirming axoneme staining specificity. Arl13b (green) also localised with EpCAM^+^ (grey) ductular reaction/LPCs in an identical staining pattern to α-acetylated tubulin. Note the absence of Arl13b expression on adjacent EpCAM^+^ cells differentiating into hepatocytes. Confocal microscopy, 63x objective. Pc (black arrow) were also detected at the cirrhotic interface in human ALD tissue using TEM. Cells expressing Pc via TEM were not biliary cells or HSCs/myofibroblasts. **(C)** Quantitation of ductular reaction/LPC (EpCAM^+^ or pan-CK^+^) Pc^+^ cells vs. ductular reaction/LPC Pc^-ve^ cells per FOV (*n* = 3 ALD samples, 10 FOV/sample). Percentage of LPCs (EpCAM^+^ or pan-CK^+^) with Pc in donor or ALD liver vs. vimentin^+^ HSCs (*n* = 3 patients/condition). Two-tailed *t-test*; ***p = 0.0001; ****p<0.0001.

In order to define canonical Hh signaling, we examined human cirrhotic liver tissue for Pc structures. Pc were readily identified at the epithelial-mesenchymal interface in human cirrhotic liver disease (Fig 3A). It is well known that mature bile ducts express Pc [29], however Pc structures were also identified on immature EpCAM^+^ cells comprising the ductular reaction and believed to contain LPCs, in diverse human liver tissues including donor, ALD, NASH and PBC human samples (Fig 3A). A secondary axoneme marker (Arl13b), was also shown to co-localise with α-acetylated tubulin, corroborating the correct identification of the structure (Fig 3B). Arl13b staining was readily identifiable on EpCAM^+^ ductular reactions/LPCs in a similar pattern to α-acetylated tubulin (Fig 3B). We duly note that LPCs are a heterogeneous cell population, and that no current gold-standard marker in humans is capable of singularly identifying LPCs without co-staining either biliary or hepatocyte epithelium. Nevertheless, the obvious anatomical differences outlined earlier between a mature human bile duct and LPCs/the ductular reaction allowed us to confirm the presence of Pc on cells morphologically representing LPCs/ductular reaction. Furthermore, a second gold standard LPC marker (pan-CK) was used to confirm the presence of Pc on ductular reactions/LPCs (S2B Fig). Pc structures were also confirmed on non-biliary, non-HSC cells located within the fibrotic septae in human ALD tissue using TEM (Fig 3B, arrow).

To demonstrate the importance of these structures within the LPC/ductular population, we quantitated the presence of Pc on LPCs/ductular reaction (EpCAM^+^ or pan-CK^+^). Single, fully formed Pc structures were found on 58.6% LPCs in donor liver (162 Pc, 279 EpCAM+ cells, *n* = 3 samples) and 42.11% LPCs in ALD liver (566 Pc, 1259 EpCAM^+^ or pan-CK cells, *n* = 3 samples), respectively (Fig 3C). Further, α-acetylated tubulin staining was found consistently associated with the ductular reaction (90% of LPCs; data not shown), even though single Pc structures were not resolved on every cell.

Intuitively, a cell reabsorbs its Pc in order to enter the cell cycle. Interestingly, we were able to show that Pc^+^ LPCs/ductular reaction (white dashed arrows) are adjacent to ‘intermediate hepatocytes’ (yellow solid arrows) (S3A Fig) which are indeed Pc^-^ and interestingly often proliferating (ki67^+^) (S3B Fig). Furthermore, the vast majority of cells within the ductular reaction in our samples were not proliferating (S3B Fig).

**Fig 4:**
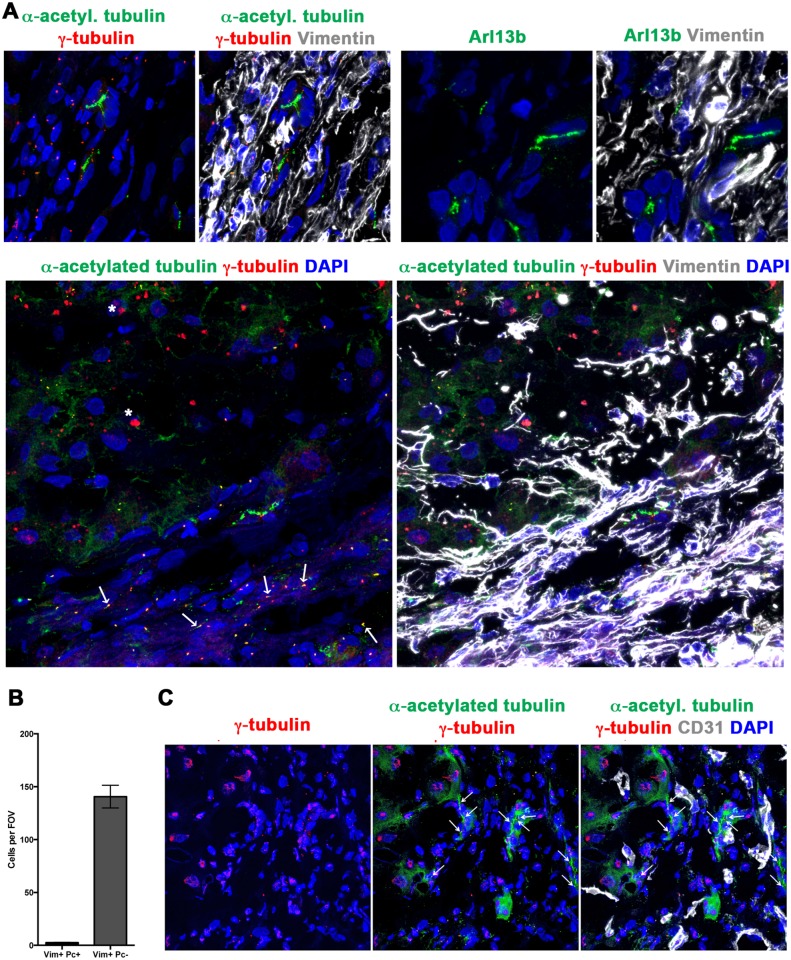
A very minimal population of human vimentin^+^ HSCs/myofibroblasts express a primary cilium, with none detected on CD31^+^ endothelial cells. Human ALD liver tissue was examined for the expression of primary cilia (α-acetylated tubulin, green; γ-tubulin, red) by vimentin^+^ (grey) HSCs/myofibroblasts **(A)** or CD31+ (grey) ECs **(C)**. **(A)** The majority of vimentin^+^ cells were Pc^-ve^ in the tissues examined. Representative image shown, displaying absence of Pc on vimentin^+^ cells. To confirm this result, ciliary protein Arl13b (green) was co-stained with vimentin (grey). Rare Arl13b ciliary structures (arrow) co-localised with vimentin^+^ cells. Final panel in A illustrates rare Pc^+^ (α-acetylated tubulin, green; γ-tubulin, red) vimentin^+^ (grey) HSCs/myofibroblasts, at the cirrhotic interface. **(B)** Number of vimentin^+^ Pc^+^ cells or vimentin^+^ Pc^neg^ cells per FOV (*n* = 3 ALD samples, 8 FOV/sample). **(C)** No Pc were detected on CD31^+^ cells in the tissues examined (ALD *n* = 3, 8 FOV/sample). Representative image shown. All images obtained using confocal microscopy, 63x objective. DAPI, blue. White arrows illustrate Pc. * Non-specific liver autofluorescence.

Only 1.92% of vimentin^+^ HSCs/myofibroblasts in chronic liver injury expressed Pc, although 99% of vimentin^+^ cells were GLI2^+^ (Figs 2E, 3C, 4A and 4B). Axoneme staining for Arl13b suggested the presence of rare vimentin^+^ cells that express a fully assembled Pc (Fig 4A, arrow). When screening for Pc, rare Pc^+^ vimentin^+^ cells were detected in ALD chronic liver injury (Fig 4A). Pc were not detected on CD31^+^ ECs in the ALD samples screened (*n* = 3; Fig 4C, representative image). Further, absence of Pc on leukocytes (CD45^+^) (*n* = 1 sample screened; data not shown) and hepatocytes assisted to validate the specificity of our staining, with these cell types previously established to be Pc^-ve^ [30].

This suggests that in human liver, the ductular/LPC niche is supported either by hepatocyte-derived (paracrine) or ductular/LPC-derived (autocrine) canonical Hh signals, with Hh ligand capable of binding to the EpCAM^+^ Pc^+^ GLI2^+^ Hh-responsive ductular/LPC population. This appears to occur frequently within liver homeostasis and in a chronic injury context.

### Effect of GLI inhibition on LPC function and gene expression

**Fig 5:**
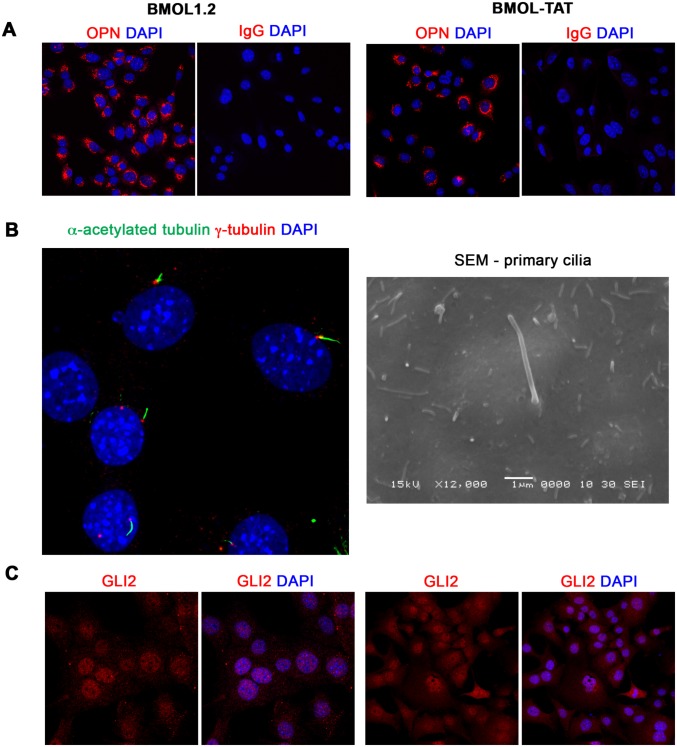
Characterisation of mouse liver progenitor cell lines. **(A)** Mouse liver progenitor cell (LPC) lines (BMOL1.2, BMOL-TAT) were stained using immunofluorescence to determine expression of LPC marker Osteopontin (OPN) (red). IgG isotype control images obtained using identical imaging conditions. DAPI, blue. **(B)** LPCs (BMOL1.2) were shown to express full-length primary cilium (Pc) structures by immunofluorescence detection of axoneme (α-acetylated tubulin, green) and basal body (γ-tubulin, red) markers. This was also confirmed by scanning electron microscopy (SEM). BMOL-TAT cells were also confirmed to express Pc via immunofluorescence and EM studies (data not shown). **(C)** Nuclear GLI2 (red) expression in LPC lines (BMOL1.2, BMOL-TAT) via immunofluorescence staining. DAPI, blue. Confocal microscopy, 63x objective.

Our mouse and human *in vivo* data provide robust evidence for active GLI signaling within many intrahepatic cell populations including LPCs [7]. We have previously ascertained that GLI signaling can be driven either canonically (Pc/SMO-dependent) or non-canonically (Pc/SMO-independent) in the mouse LPC line BMOL1.2 [7]. Further, SMO inhibition (or hGLI3-R overexpression) significantly decreases LPC proliferation/viability, whilst hGLI1 overexpression significantly increases proliferation/viability [7]. To further explore the nature of GLI signaling within the LPC and to determine the effect of GLI inhibition on LPC proliferation and gene expression, we used the two well-characterized LPC lines BMOL1.2 and BMOL-TAT [22]. Characterisation of LPC-specific marker OPN [24], Pc and GLI expression within these cell lines is provided in Fig 5.

**Fig 6:**
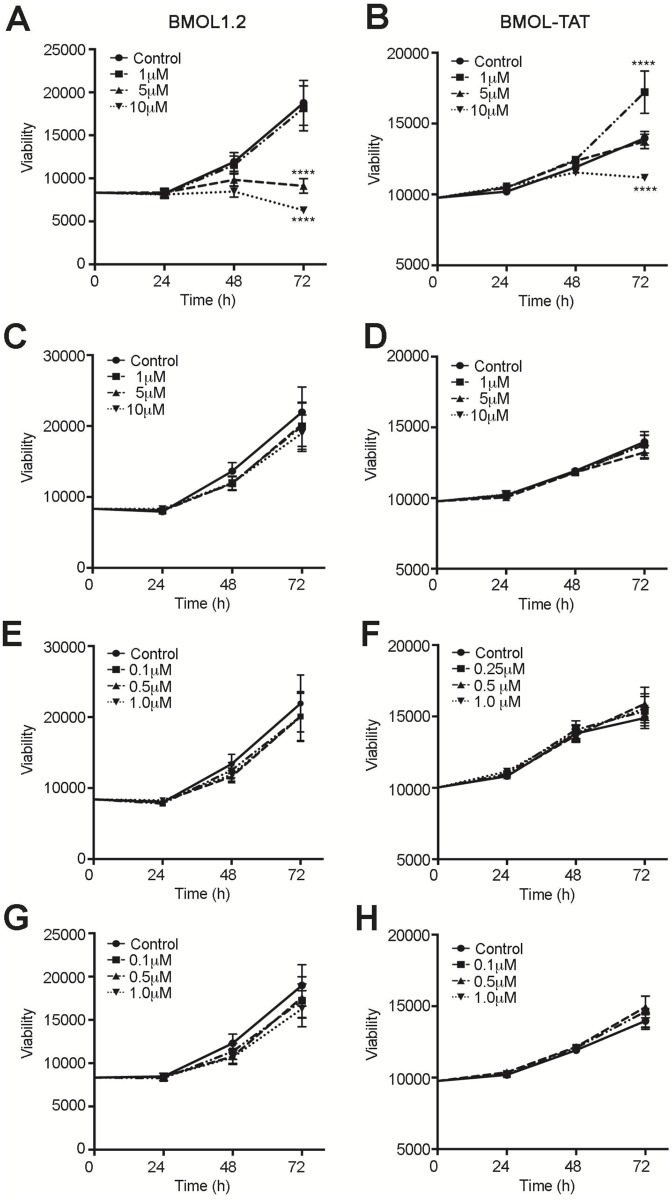
Inhibition of GLI signaling reduces the viability of liver progenitor cell lines. LPC lines BMOL1.2 **(A, C, E, G)** and BMOL-TAT **(B, D, F, H)** were grown over 72 h in the presence of signaling inhibitors for GLI (GANT61; **A, B**), Notch (DAPT; **C,D**) c-Met (SGX523; **E, F**), Wnt (XAV939; **G, H**) signaling and matching [DMSO]. Viability was determined by ALAMAR Blue assays, and an interaction between time and dose was assessed using two-way ANOVA followed by Tukey’s multiple comparisons test (α = 0.001) (Prism, GraphPad). **** *p*<0.001.

Both BMOL1.2 and BMOL-TAT cells showed a significant reduction in viability following addition of 10 μM of specific GLI inhibitor GANT61 (GLI1, GLI2) compared to matched DMSO controls (*p*<0.001) in three separate experiments (Fig 6A). A difference between the two LPC lines was observed following addition of low concentration GANT61 (1 μM), which significantly increased cell viability in BMOL-TAT cells (*p*<0.001) compared to controls. Interestingly, medium supplementation with active concentrations of Notch, c-Met and Wnt inhibitors had no effect on BMOL1.2 or BMOL-TAT cell viability, within a 72 h time frame (Fig 6C–6H).

**Fig 7:**
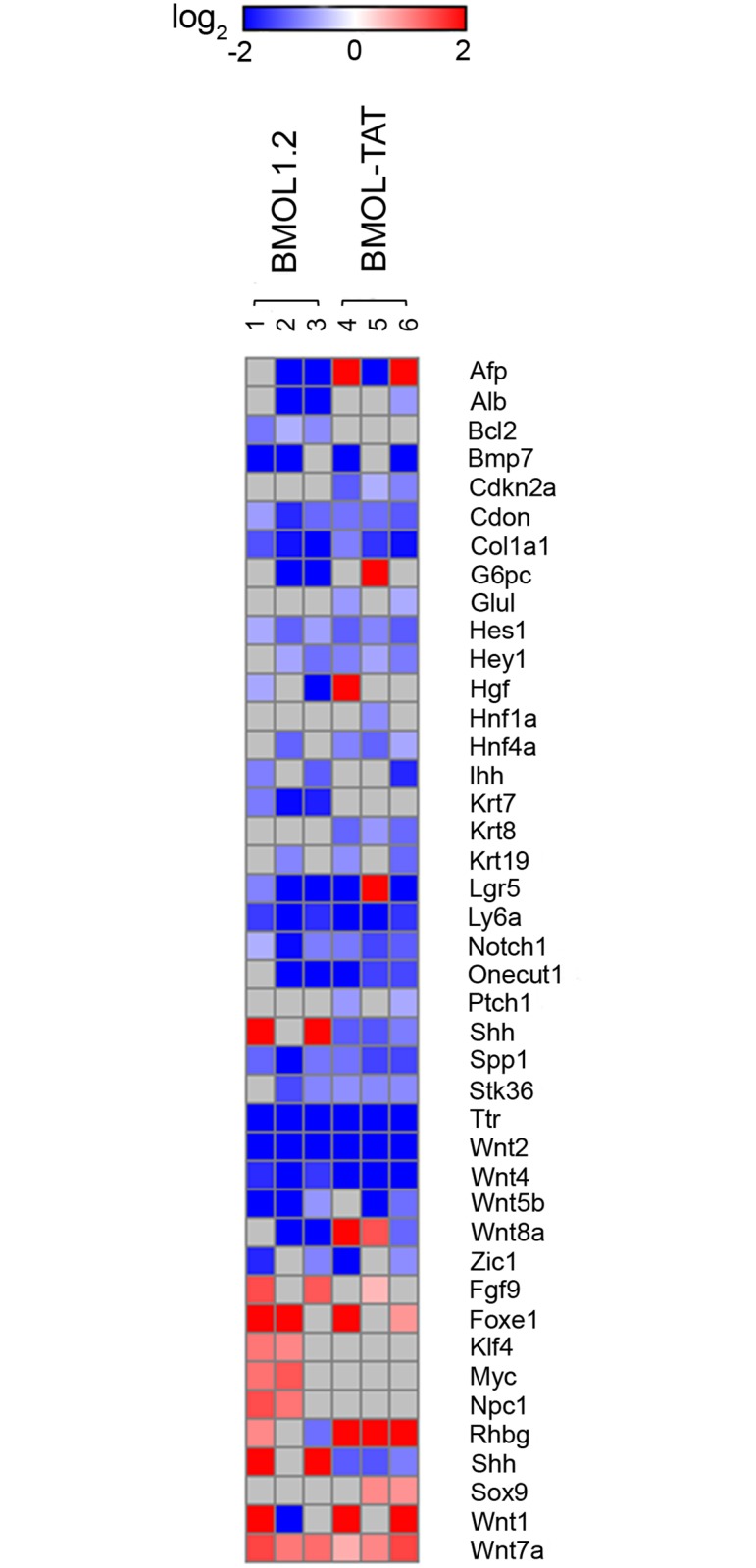
Changes to transcript expression in two liver progenitor cell lines following GLI inhibition. Heat map representing changes to gene transcript levels in BMOL1.2 (*n* = 3) or BMOL-TAT (*n* = 3) lines following GANT61 (10 μM) 8 h treatment. Log_2_ intensity scale shown. Downregulated genes (blue) are shown above the yellow dashed line, with upregulated genes (red) below. Grey indicates no data.

In order to determine the effect of GLI inhibition on gene transcription in these cells, a custom PCR array platform was designed to interrogate 89 genes associated with Hh, Wnt, and Notch signaling, as well as proliferation and differentiation status. A dose of 10 μM GANT61 was chosen as this dose had similar effects in both cell lines. Major gene changes are displayed in Tables 2 and 3. Importantly, specific inhibition of GLI resulted in similar gene expression changes in both BMOL1.2 and BMOL-TAT lines (Fig 7).

**Table 2:** Gene changes in BMOL1.2 cells following GANT61 treatment.

Gene ID	Exp 1	Exp 2	Exp 3	Av. fold change	P-value
Wnt7a	2.8128	2.1124	2.2579	2.3761	**0.004657**
Npc1	2.6938	2.1547		2.0175	**0.020898**
Klf4	2.1368	1.9517		1.7288	**0.033877**
Myc	2.1844	2.548		1.8816	**0.045375**
Shh	8.8644		16.916	5.1014	0.068148
Foxe1	14.8298	8.9489		5.4696	0.250029
Fgf9	2.6993		2.5142	1.7262	0.577767
Frmd6	1.8428	1.5519		*	*
Ttr	-13.2214	-17.2598	-7.472	-11.9468	**0.000012**
Wnt4	-3.1182	-6.6949	-2.9473	-3.9478	**0.001959**
Cdon	-1.6804	-3.1941	-2.2437	-2.2922	**0.00247**
Hes1	-1.5784	-2.3333	-1.6731	-1.8333	**0.004203**
Wnt5b	-24.0962	-9.3581	-1.7676	-7.3593	0.008741
Col1a1	-2.5565	-3.5912	-21.1182	-5.7878	**0.013104**
Ly6a	-2.8764	-6.6761	-3.1031	-3.9059	**0.013663**
Notch1	-1.5275	-3.8129	-2.0159	-2.2729	**0.015099**
Spp1	-2.2784	-4.5974	-2.0979	-2.8009	**0.022118**
Krt7	-2.023	-3.797	-3.3846	-2.9624	**0.041302**
Bcl2	-2.0901	-1.5318	-1.8685	-1.8153	0.080586
Wnt2	-5.8314	-169.6059	-35.4194	-32.7205	0.127555
Lgr5	-1.9422	-101.6917	-37.5388	-19.4992	0.192774
Stk36		-2.6693	-1.9135	-1.9399	**0.005156**
Hey1		-1.6072	-2.173	-1.639	0.064545
Alb		-4.4566	-4.7849	-2.9446	0.070115
Wnt8a		-9.5955	-4.4963	-3.9738	0.070416
Onecut1		-8.8591	-4.6342	-3.4717	0.093108
G6pc		-53.1383	-32.2536	-13.559	0.12305
Afp		-116.1394	-22.7099	-15.6542	0.161233
Ihh	-1.9849		-2.3811	-1.5854	0.2345
Bmp7	-54.3537	-25.573		-10.4076	0.251635
Zic1	-3.2311		-1.94	-1.9514	0.325106
Hgf	-1.5956		-10.9219	-2.807	0.39019

* Unable to be determined in statistical analysis

**Table 3:** Gene changes in BMOL-TAT cells following GANT61 treatment.

Gene ID	Exp 1	Exp 2	Exp 3	Av. fold change	P-value
Rhbg	11.1573	33.3409	81.1833	31.1411	**0.00654**
Sox9		1.9169	1.834	1.7193	0.067694
Wnt7a	1.5741	1.9703	2.8059	2.0569	0.165904
Wnt1	10.7952		22.9525	5.5068	0.167538
Foxe1	25.7235		1.7892	3.142	0.3836
Wnt3		1.6958	3.2958		
Cdon	-2.1262	-2.1908	-2.4479	-2.2508	**0.000078**
Hes1	-2.3578	-1.9124	-2.4136	-2.216	**0.000364**
Wnt4	-7.9792	-4.1325	-4.9943	-5.4813	**0.000914**
Onecut1	-3.9252	-2.7838	-2.6935	-3.0875	**0.006028**
Stk36	-1.8248	-1.8813	-1.8581	-1.8546	**0.006206**
Hey1	-1.9619	-1.6142	-2.0272	-1.8586	**0.017316**
Ly6a	-4.7474	-4.4984	-2.9875	-3.9958	**0.017748**
Krt8	-2.2808	-1.7486	-2.2377	-2.0742	**0.026401**
Ttr	-289.9103	-4.6421	-5.4956	-19.4834	**0.029609**
Spp1	-2.1086	-2.7616	-2.7243	-2.5127	0.051541
Notch1	-2.0518	-2.7129	-2.3918	-2.3701	0.06564
Col1a1	-1.9938	-3.014	-3.6769	-2.8061	0.087278
Hnf4a	-1.9321	-2.3152	-1.5938	-1.9247	0.1736
Wnt2	-31.9845	-63.6089	-14.3771	-30.8113	0.20457
Cdkn2a	-2.408	-1.526	-1.9382	-1.924	0.324337
Shh	-2.4149	-2.4826	-2.0008	-2.2891	0.379243
Ptch1	-1.7314		-1.5856	-1.5262	**0.030954**
Glul	-1.7141		-1.5636	-1.555	0.099958
Hnf1a	-1.6511	-1.8324		-1.5689	0.169875
Krt19	-1.8281		-2.2316	-1.8149	0.218108
Bmp7	-7.3819		-5.0683	-3.8147	0.288082
Zic1	-6.3107		-1.8402	-2.0463	0.308904
Wnt5b		-8.6256	-2.1608	-3.3145	0.336567
Disp1	-1.6681	-1.5164			

Downregulation of genes was more frequent than upregulation, when blocking GLI-dependent transcription with GANT61 in BMOL1.2 (24/89 down; 8/89 up) (Table 2) and BMOL-TAT (24/89 down; 6/89 up) (Table 3) cell lines. Downregulation of *Bmp7* and *Zic1* occurred in 2/3 experiments in both cell lines, while cell line-specific GANT61-dependent downregulation was also observed in transcripts of *Afp*, *Alb*, *Bcl2*, *G6pc*, *Hgf*, *Ihh*, *Krt7*, *Wnt8a* (BMOL1.2), and *Cdkn2a*, *Glu1*, *Hnf1a*, *Hnf4a*, *Krt8*, *Krt19*, *Ptch1* (BMOL-TAT). In order to identify common changes in LPC genes in response to GANT61 a combination analysis of all six experiments was performed, in which GANT61 reduced expression by >1.5-fold in 14/89 genes (*Cdon*, *Col1a1*, *Hes1*, Hey1, *Lgr5*, *Ly6a*, *Notch1*, *Onecut1*, *Spp1*, *Stk36*, *Ttr*, *Wnt2*, *Wnt4*, *Wnt5b*) in 5/6 array experiments (Table 4). Further, downregulation of *Alb*, *Bmp7*, *Cdon*, *Col1a1*, *Hes1*, *Hey1*, *Krt8*, *Ly6a*, *Notch1*, *Onecut1*, *Spp1*, *Stk36*, *Ttr*, *Wnt2*, *Wnt4*, *Wnt5b* genes was statistically significant (Table 4), of which *Wnt2*, *Ttr* and *Bmp7* had the greatest reduction of transcript levels on average in response to GANT61 (-31.75, -15.26, and -6.30 fold respectively).

**Table 4:** Common gene changes in BMOL1.2 and BMOL-TAT cells following GANT61 treatment.

	BMOL1.2					BMOL-TAT					COMBINED ANALYSIS	
*Gene ID*	1	2	3	$\bar{\text{X}}$ fold change	*p*-value	4	5	6	$\bar{\text{X}}$ fold change	*p*-value	$\bar{\text{X}}$ fold change	*p*-value
*Afp*		-116.1	-22.7	-15.65	0.1612	14.1	-38.0	40.3	-	-	-2.52	0.45926
*Alb*		-4.5	-4.8	-2.94	**0.0701**			-1.7	-	-	-2.06	**0.02770**
*Bcl2*	-2.1	-1.5	-1.9	-1.82	**0.0806**				-	-	-	-
*Bmp7*	-54.4	-25.6		-10.41	0.2516	-7.4		-5.1	-3.815	0.288082	-6.30	**0.09271**
*Cdkn2a*				-	**-**	-2.4	-1.5	-1.9	-1.924	0.324337	-1.54	0.18022
*Cdon*	-1.7	-3.2	-2.2	-2.29	**0.0025**	-2.1	-2.2	-2.4	-2.251	**<0.0001**	-2.27	**<0.0001**
*Col1a1*	-2.6	-3.6	-21.1	-5.79	**0.0131**	-2.0	-3.0	-3.7	-2.806	**0.087278**	-4.03	**0.00101**
*G6pc*		-53.1	-32.3	-13.56	**0.1231**		28.2		-	-	-2.52	0.51636
*Glul*				-	**-**	-1.7		-1.6	-1.555	**0.099958**	-	-
*Hes1*	-1.6	-2.3	-1.7	-1.83	**0.0042**	-2.4	-1.9	-2.4	-2.216	**0.000364**	-2.02	**0.00032**
*Hey1*		-1.6	-2.2	-1.64	**0.0645**	-2.0	-1.6	-2.0	-1.859	**0.017316**	-1.75	**0.00079**
*Hgf*	-1.6		-10.9	-2.81	0.3902	5.8			-	-	-	-
*Hnf1a*				-	-	-1.7	-1.8		-1.569	0.169875	-	-
*Hnf4a*		-2.3		-	-	-1.9	-2.3	-1.6	-1.925	0.1736	-1.74	0.12746
*Ihh*	-2.0		-2.4	-1.59	0.2345			-3.2	-	-	-1.83	0.23362
*Krt7*	-2.0	-3.8	-3.4	-2.96	**0.0413**				-	-	-1.91	0.60786
*Krt8*				-	**-**	-2.3	-1.7	-2.2	-2.074	**0.026401**	-1.56	**0.08193**
*Krt19*		-1.9		-	**-**	-1.8		-2.2	-1.815	0.218108	-1.60	0.29123
*Lgr5*	-1.9	-101.7	-37.5	-19.50	0.1928	-6.2	31.3	-48.6	-	-	-6.45	0.13103
*Ly6a*	-2.9	-6.7	-3.1	-3.91	**0.0137**	-4.7	-4.5	-3.0	-3.996	**0.017748**	-3.95	**0.00302**
*Notch1*	-1.5	-3.8	-2.0	-2.27	**0.0151**	-2.1	-2.7	-2.4	-2.370	**0.06564**	-2.32	**0.00661**
*Onecut1*		-8.9	-4.6	-3.47	**0.0931**	-3.9	-2.8	-2.7	-3.088	**0.006028**	-3.27	**0.00130**
*Ptch1*				-	**-**	-1.7		-1.6	-1.526	**0.030954**	-	-
*Shh*	8.9		16.9	-	**-**	-2.4	-2.5	-2.0	-2.289	0.379243	-	-
*Spp1*	-2.3	-4.6	-2.1	-2.80	**0.0221**	-2.1	-2.8	-2.7	-2.513	**0.051541**	-2.65	**0.07831**
*Stk36*		-2.7	-1.9	-1.94	**0.0052**	-1.8	-1.9	-1.9	-1.855	**0.006206**	-1.90	**<0.0001**
*Ttr*	-13.2	-17.3	-7.5	-11.95	**<0.0001**	-289.9	-4.6	-5.5	-19.483	**0.029609**	-15.26	**<0.0001**
*Wnt2*	-5.8	-169.6	-35.4	-32.72	0.1276	-32.0	-63.6	-14.4	-30.811	**0.20457**	-31.75	**0.07108**
*Wnt4*	-3.1	-6.7	-2.9	-3.95	**0.0020**	-8.0	-4.1	-5.0	-5.481	**0.000914**	-4.65	**<0.0001**
*Wnt5b*	-24.1	-9.4	-1.8	-7.36	**0.0087**		-8.6	-2.2	-3.315	0.336567	-4.94	**0.02858**
*Wnt8a*		-9.6	-4.5	-3.97	**0.0704**	5.6	2.6	-2.3	-	-	-	-
*Zic1*	-3.2		-1.9	-1.95	0.3251	-6.3		-1.8	-2.046	0.308904	-2.00	0.11378
*Fgf9*	2.7		2.5	1.73	0.5778		1.5		-	-	1.53	0.36967
*Foxe1*	14.8	8.9		5.47	0.2500	25.7		1.8	3.142	0.3836	4.15	0.18450
*Klf4*	2.1	2.0		1.73	**0.0339**				-	-	-	-
*Myc*	2.2	2.5		1.88	**0.0454**				-	-	1.51	0.20080
*Npc1*	2.7	2.2		2.02	**0.0209**				-	-	-	-
*Rhbg*	1.9		-2.2	-	**-**	11.2	33.3	81.2	31.1411	**0.00654**	5.45	0.39536
*Shh*	8.9		16.9	5.10	**0.0681**	-2.4	-2.5	-2.0	-	-	-	-
*Sox9*				-	**-**		1.9	1.8	1.7193	**0.067694**	1.51	**0.01519**
*Wnt1*	13.3	-56.2		-	**-**	10.8		23.0	5.5068	0.167538	1.91	0.45073
*Wnt7a*	2.8	2.1	2.3	2.38	**0.0047**	1.6	2.0	2.8	2.0569	0.165904	2.21	**0.00326**

While *Wnt7a* and *Foxe1* genes were upregulated in both cell lines in response to GANT61, cell line- specific transcript upregulation also occurred in BMOL1.2 (*Fgf9*, *Klf4*, *Myc*, *Npc1*) and BMOL-TAT (*Rhbg*, *Wnt1*) cells in 2/3 experiments (Table 4). Interestingly, *Shh* was increased in BMOL1.2 but decreased in BMOL-TAT in response to GANT61. Also, >1.5-fold *Sox-9* transcript upregulation was observed in 2/3 BMOL-TAT experiments only, however, combinatorial analysis of both cell lines determined that a statistically significant upregulation of *Sox-9* transcript was evident (1.51-fold; *p* = 0.015) as well as a 2.21-fold increase in *Wnt7a* (*p* = 0.0033).

## Discussion

The key observations in this paper include the finding that canonical Hh (Pc/SMO-dependent, GLI-mediated signaling) is predominately restricted to the EpCAM^+^ ductular compartment in human liver tissue. In contrast, GLI-mediated signals appear to be Pc/SMO-independent within multiple Pc^-ve^ GLI2^+^ human intrahepatic compartments (namely, HSC, hepatocyte, leukocyte and endothelial). Other novel aspects of this study include the observation that GLI delivers a pro-survival signal to LPCs in culture. An obvious caveat to our work is our key observations are mostly descriptive and based on a small sample size of human liver specimens. Nevertheless, our results provide the first methodical and comprehensive assessment of Pc/GLI expression in human liver disease specimens, and identify that GLI signals are mediated at least via two mechanisms in CLD. This has immediate translational significance: anti-GLI therapies (possibly in combination with anti-SMO) may be a better Hh target for novel CLD therapies, currently in stage I trials.

The impact of this study is several-fold. Firstly, we have made the novel discovery that Pc are expressed by LPCs (EpCAM^+^, pan-CK^+^) in human liver tissue—both in donor and cirrhotic liver of several etiologies (ALD, NASH, PBC). This paper provides the first evidence that Pc-mediated signaling pathways e.g. Hh (and possibly others yet to be identified) are of importance/biologically significant in human liver tissue pertaining to regulation of the LPC niche. These data have not been reported previously, with our group being the first to identify Pc on LPCs in mouse liver tissue in 2014 [7]. The presence of Pc on normal intrahepatic bile ducts has long been appreciated. Yet Pc-regulated signaling has not previously been studied within the context of the human LPC niche, although many stem cell niches in other organ systems e.g. brain [31], muscle [32], lung [33], and also embryonic stem cells [34], are known to be regulated (at least in part) via Pc-mediated signals. Future studies are required to understand the contribution of Pc-mediated signaling pathways to the evolution of the human LPC niche from homeostasis to chronic injury, as gaining these insights will most likely have important ramifications to gaining understanding in to how liver cancer progresses.

Secondly, we have identified gene changes in BMOL1.2 and BMOL-TAT LPC lines that occur with GLI inhibition (discussed below). Thirdly, we have validated in human tissue samples that GLI-mediated signals are mediated by at least two mechanisms in chronic liver injury; this has important ramifications for current clinical trials, and is discussed in greater detail below. Fourthly, we have provided additional verification to prior studies pertaining to characterising Hh-producing and Hh-responding cell populations in human chronic liver disease, which is required to strengthen the field.

Our *in vitro* culture studies provide further support to our prior studies [7] and human data, and strongly suggest the GLI transcriptome is a critical pathway in LPC biology. Inhibition of GLI saw a similar downregulation of a defined gene set within the two LPC lines. In general, three main gene groups appear to be GLI-dependent: stem cell genes, hepatocyte/biliary differentiation genes, and Hh/Wnt pathway genes.

Stem cell genes inhibited by GANT61 include *Lgr5*, *Hes1*, *Hey1*, *Ly6a*, *Wnt2*, *Wnt4*, and *Wnt5b* transcripts. *Lgr5* is a well-known marker of proliferating Wnt-driven stem cells [35] and marks proliferating murine LPCs *in vivo* [35]. *Lgr5* was downregulated by up to 100-fold. Likewise, *Wnt2* was highly downregulated (35- to 150-fold). Furthermore, two of the four chief pluripotency genes (*Klf4*, *myc*) were upregulated in BMOL1.2 cells following GANT61 treatment, concurrent with an increase in multiple Hh-related genes (*Fgf9*, *Foxe1*, *Npc1*, *Shh)*. This potentially demonstrates a compensation event, if GLI signals are indeed maintaining LPC stemness and survival. Overall, our results imply that GLI is involved in maintaining stemness in the LPC lines. We hypothesise from our data that GLI delivers a pro-survival signal to LPCs whilst maintaining their stemness, subsequently allowing for appropriate hepatocyte (Wnt-driven) and biliary (Notch-driven) differentiation responses [36].

The major findings of this study confirm our prior observations in the TAA model [7] that GLI signaling in CLD is mediated by at least two mechanisms: Pc/SMO-dependent and Pc/SMO-independent GLI-mediated signaling. The interpretation of these data has important ramifications, given anti-Hh therapy is currently being used as a novel target for hepatocellular carcinoma and cirrhosis treatment (presently in stage I clinical trials [13]). Importantly, current trials have selected an anti-SMO inhibitor (LDE225) for cirrhosis treatment; however, our results suggest that GLI (either with or without combinatorial targeting of SMO) may be a more appropriate signaling node within the Hh pathway to target. In support of our observations, mouse fibrosis intervention studies in the lung [37] and kidney [38] have indicated that anti-GLI rather than anti-SMO inhibitors are effective at decreasing fibrosis—and indicate that GLI signaling is (at least partially) controlled via SMO-independent mechanisms in these models. Prior studies in mouse liver injury models have indicated that deletion of SMO in HSCs decreases the degree of fibrosis [39]. However, in light of our observations (both in the TAA mouse model and now confirmed in human liver tissue samples), combinatorial targeting of SMO/GLI should be tested in liver injury models, and anti-GLI inhibitors considered for the design of clinical trials involving anti-Hh therapies for the treatment of CLD.

To conclude, this work provides the first thorough assessment of Pc/GLI expression in human liver disease, and confirms (following our initial observations in the mouse) that GLI signals are likely to be mediated via at least two mechanisms in CLD. These results have immediate translational significance and should be considered prior to initiating stage II clinical trials using SMO inhibitors.

## Supporting information

S1 Fig IgG controls for immunofluorescence. **(A)** As a negative control for immunofluorescence studies, rabbit IgG (SHH, ab53281) was substituted at the same final concentration as the primary antibody listed. 5x objective. IgG controls are also shown for the SHH confocal microscopy studies in Fig 1, obtained using identical imaging conditions at the 63x objective. DAPI, blue. No specific staining was observed. **(B)** As a negative control for immunofluorescence studies, rabbit IgG (GLI2, ab7181) was substituted at the same final concentration as the primary antibody listed. 5x objective. IgG controls are also shown for the GLI2 confocal microscopy studies in Fig 2, obtained using identical imaging conditions at the 63x objective. DAPI, blue. No specific staining was observed.
(TIF)

S2 Fig SHH expression by hepatocytes in human NASH, and primary cilia expression by pan-CK^+^ liver progenitor cells. **(A)** SHH (red) was expressed by CK18^+^ (green) hepatocytes in human NASH samples. **(B)** Human ALD liver tissue samples were examined for primary cilium expression (α-acetylated tubulin, green; γ-tubulin, red) using a second liver progenitor cell (LPC) marker, pan-cytokeratin (pan-CK)^+^ (grey). Pan-CK^+^ cells also expressed Pc. DAPI, blue. All images obtained using confocal microscopy, 63x objective.
(TIF)

S3 Fig Liver progenitor cells expressing Pc are often adjacent to proliferating Pc^-ve^ intermediate hepatocytes. **(A)** Human ALD liver tissue samples were examined for primary cilium expression (α-acetylated tubulin, green; γ-tubulin, red) on LPCs (pan-CK)^+^ (grey) indicated by white dashed arrows. Yellow solid arrows indicate intermediate hepatocytes found adjacent to these Pc^+^ LPCs. DAPI, blue. **(B)** LPCs (green; EpCAM or pan-CK) were co-stained with the proliferation marker ki67 (red). Intermediate hepatocytes adjacent to LPCs were often ki67^+^ (yellow arrows). All images obtained using confocal microscopy, 63x objective. * non-specific staining in ki67 channel.
(TIF)

S1 Table Custom 384-well murine gene array (CAPM12471E) layout. (XLSX)

## Abbreviations

ALD: alcoholic liver disease
CLD: chronic liver disease
DHH: Desert Hedgehog
EC: endothelial cell
FOV: field of view
Hh: Hedgehog
HSC: hepatic stellate cell
IF: immunofluorescence
IHH: Indian Hedgehog
LPC: liver progenitor cell
MIP: maximum intensity projection
NASH: non-alcoholic steatohepatitis
NBF: neutral buffered formalin
O/N: overnight
Pan-CK: pan-cytokeratin
PBC: primary biliary cirrhosis
Pc: primary cilia
RT: room temperature
SHH: Sonic Hedgehog
SMO: Smoothened
TAA: thioacetamide
TEM: transmission electron microscopy
