# Supplementary figures and images for: Widespread GLI expression but limited canonical hedgehog signaling restricted to the ductular reaction in human chronic liver disease
Source: PLoS One. 2017 Feb 10;12(2):e0171480. doi: 10.1371/journal.pone.0171480 (PMC5302813; doi:10.1371/journal.pone.0171480)

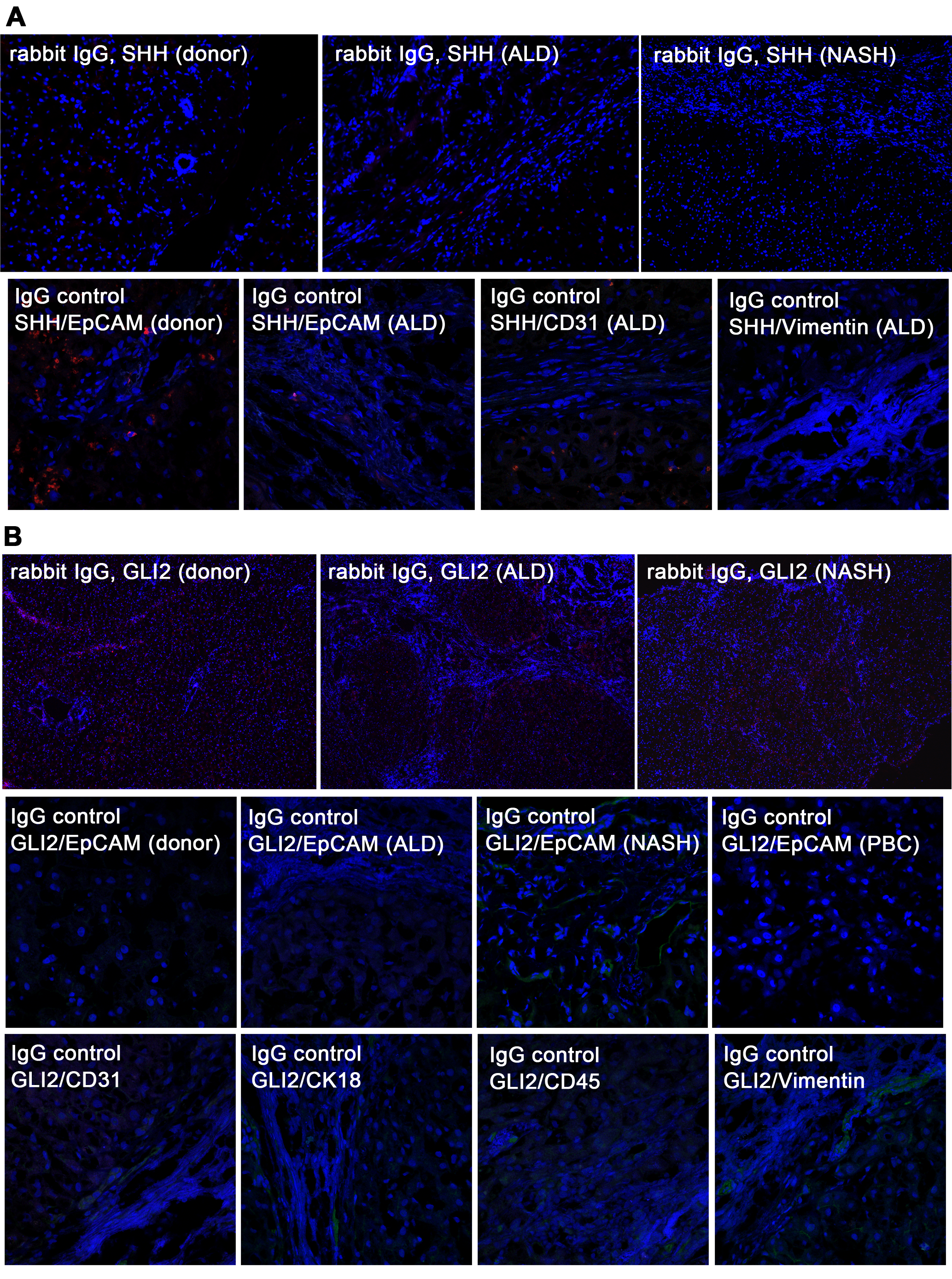

Supplement: S1 Fig — (A) As a negative control for immunofluorescence studies, rabbit IgG (SHH, ab53281) was substituted at the same final concentration as the primary antibody listed. 5x objective. IgG controls are also shown for the SHH confocal microscopy studies in Fig 1, obtained using identical imaging conditions at the 63x objective. DAPI, blue. No specific staining was observed. (B) As a negative control for immunofluorescence studies, rabbit IgG (GLI2, ab7181) was substituted at the same final concentration as the primary antibody listed. 5x objective. IgG controls are also shown for the GLI2 confocal microscopy studies in Fig 2, obtained using identical imaging conditions at the 63x objective. DAPI, blue. No specific staining was observed. (TIF) [file pone.0171480.s001.tif]

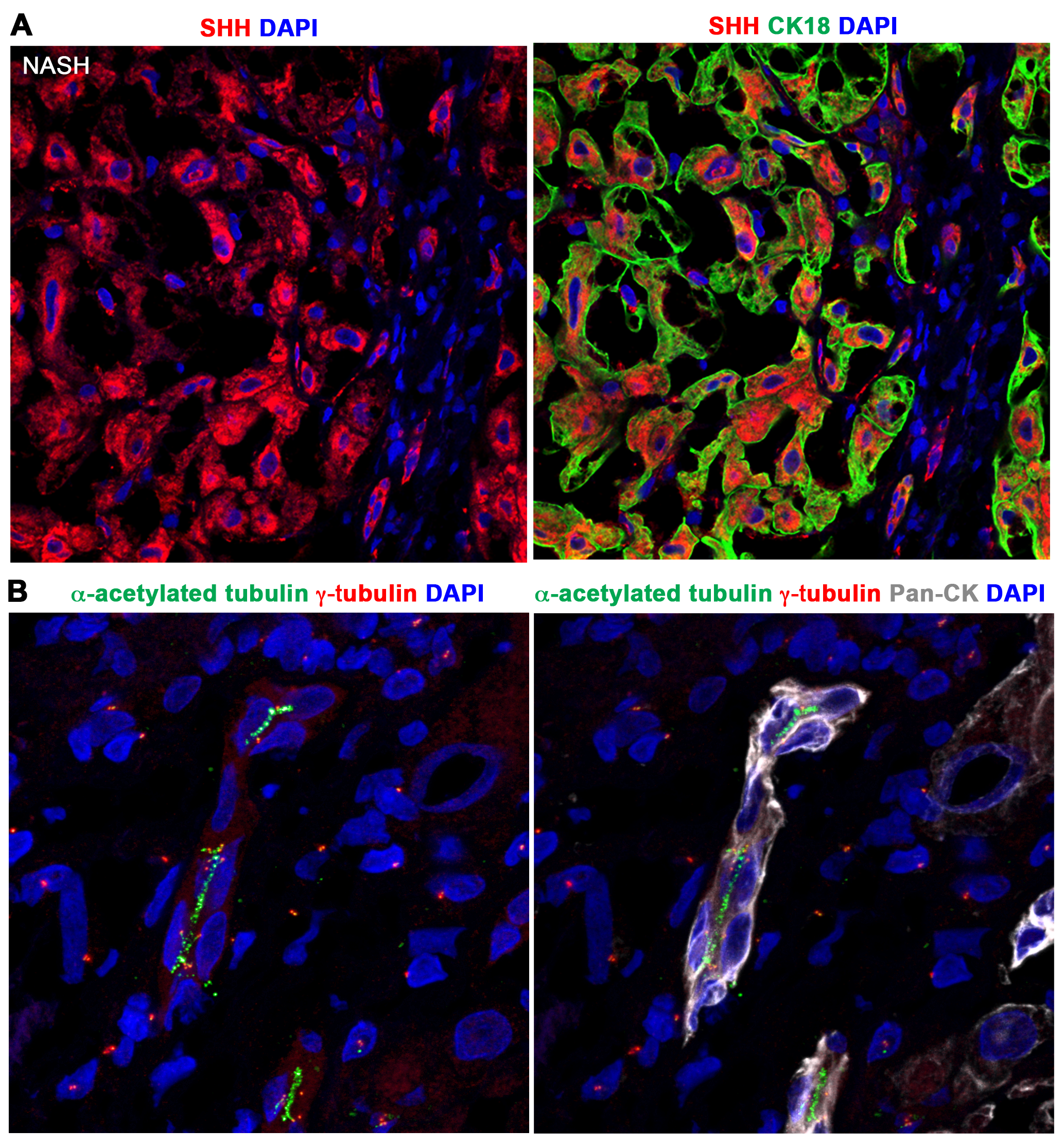

Supplement: S2 Fig — (A) SHH (red) was expressed by CK18+ (green) hepatocytes in human NASH samples. (B) Human ALD liver tissue samples were examined for primary cilium expression (α-acetylated tubulin, green; γ-tubulin, red) using a second liver progenitor cell (LPC) marker, pan-cytokeratin (pan-CK)+ (grey). Pan-CK+ cells also expressed Pc. DAPI, blue. All images obtained using confocal microscopy, 63x objective. (TIF) [file pone.0171480.s002.tif]

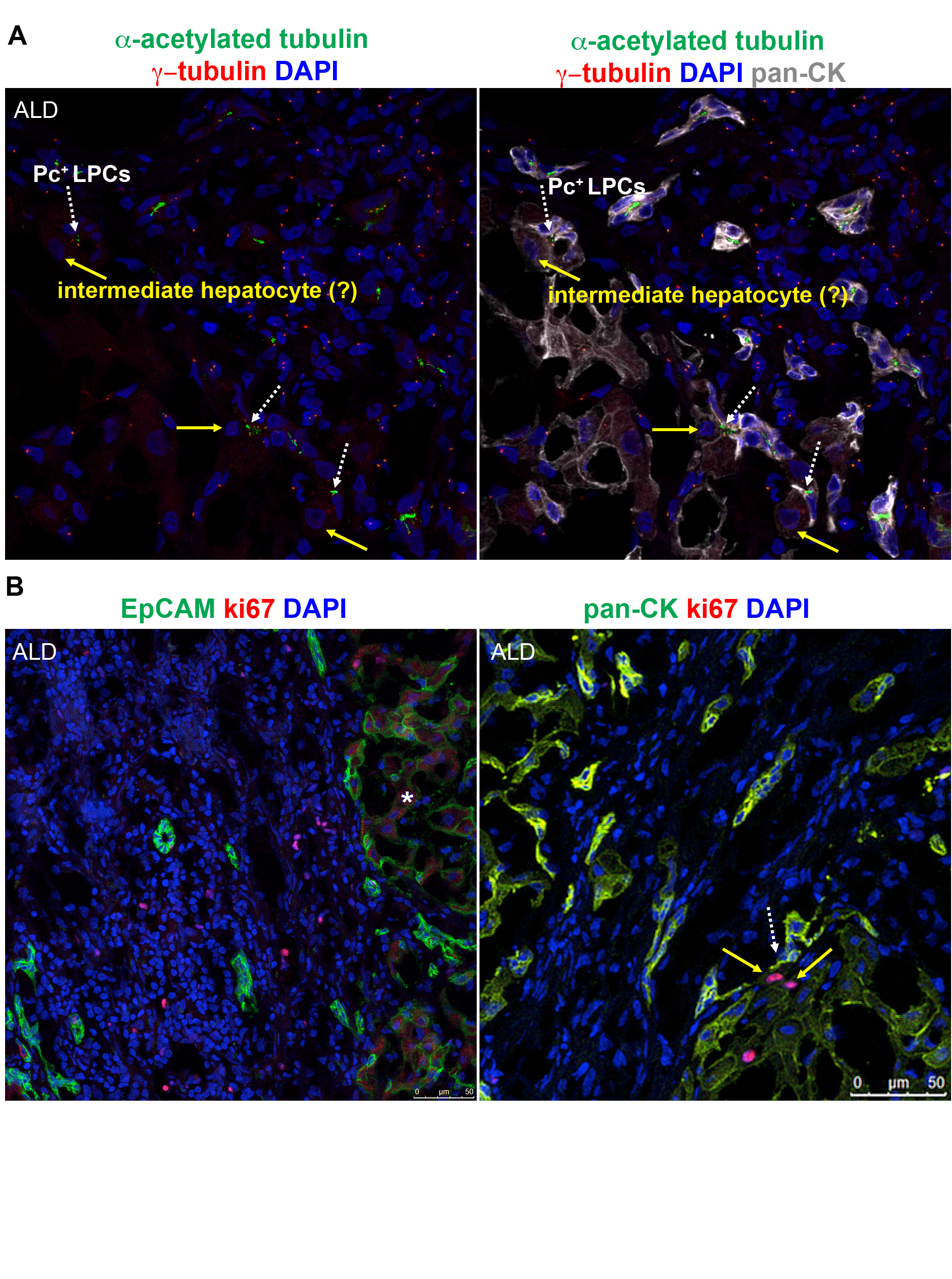

Supplement: S3 Fig — (A) Human ALD liver tissue samples were examined for primary cilium expression (α-acetylated tubulin, green; γ-tubulin, red) on LPCs (pan-CK)+ (grey) indicated by white dashed arrows. Yellow solid arrows indicate intermediate hepatocytes found adjacent to these Pc+ LPCs. DAPI, blue. (B) LPCs (green; EpCAM or pan-CK) were co-stained with the proliferation marker ki67 (red). Intermediate hepatocytes adjacent to LPCs were often ki67+ (yellow arrows). All images obtained using confocal microscopy, 63x objective. * non-specific staining in ki67 channel. (TIF) [file pone.0171480.s003.tif]
